# Evidence of drug-response heterogeneity rapidly generated from a single cancer cell

**DOI:** 10.18632/oncotarget.17064

**Published:** 2017-04-12

**Authors:** Rong Wang, Chengmeng Jin, Xun Hu

**Affiliations:** ^1^ Cancer Institute, A Key Laboratory For Cancer Prevention & Intervention, Ministry of Education of the People's Republic of China, The Second Affiliated Hospital, Zhejiang University School of Medicine, Hangzhou, China

**Keywords:** heterogeneity, cancer, drug-response

## Abstract

One cancer cell line is believed to be composed of numerous clones with different drug sensitivity. We sought to investigate the difference of drug-response pattern in clones from a cell line or from a single cell. We showed that 22 clones derived from 4T1 cells were drastically different from each other with respect to drug-response pattern against 11 anticancer drugs and expression profile of 19 genes associated with drug resistance or sensitivity. Similar results were obtained using daughter clones derived from a single 4T1 cell. Each daughter clone showed distinct drug-response pattern and gene expression profile. Similar results were also obtained using Bcap37 cells. We conclude that a single cancer cell can rapidly produce a population of cells with high heterogeneity of drug response and the acquisition of drug-response heterogeneity is random.

## INTRODUCTION

Cancer drug resistance is a major obstacle for chemotherapy. The cellular resistance to chemotherapy can be due to (1) drug extrusion mediated by drug transporter; (2) activated repair of DNA damage; (3) enhanced metabolism and detoxification of drugs; and (4) apoptosis inhibition with, in some cases, autophagy activation [1, 2].

Drug resistance can be arisen from heterogeneity of cancer cells, which exhibit differences on the aspects of growth, apoptosis, morphology, genetic instability [3, 4]. The major factors determining cancer cell heterogeneity are genetic makeup and epigenetic expression, origin of cancer stem cells, and tumor microenvironment, etc [5–10]. The advance of the technology to sequence the whole genome of a single cell has further unraveled the heterogenic nature of cancer cells [11, 12]. The intratumoral heterogeneity confounds significantly cancer therapy and is a challenge for precision medicine [13].

It was previously reported that after implantation of clones from human colorectal cancer into NOD-SCID mice for a series of generations, the clones showed five types of growth pattern. However, after oxaloplatin was given, the growth patterns changed [14]. Kim et al observed that clones derived from glioblastoma exhibited differences such as growth rate, differentiation, and response to temozolomide. And the different sensitivity in responding to temozolomide was due to the different expression of some genes responsible for drug resistance [11]. The heterogeneity also existed in lung cancer, breast cancer, AML, pancreatic cancer, prostate cancer and it significantly influence chemotherapy [6, 12, 15, 16].

Moreover, cancer cells with the same genetic makeup, i.e., daughter cells from one cell, can display fluctuation of gene expression; such fluctuation is due to randomness in evolution, namely random heterogeneity or intrinsic noise [17–20]. Spencer et al observed 2 daughter cells from one cancer cell showed a time difference in responding to TRAIL mediated apoptosis and this difference was caused by protein expression level (intrinsic noise) other than genetic or epigenetic factors [21]. Karen found that 2 daughter cells from one cell exhibited different response to antimitotic drugs; while one daughter cell died in M phase, the other went through M phase but may die at interphase or even survived; the different fates of 2 daughter cells were associated with degradation of cyclin B1 [22].

It is known that a cell line was composed of clones with different drug-response pattern, but it is not known how big the difference these clones can be. In addition, it is not known if a single cancer cell can generate an array of daughter cells with diverse drug-response phenotypes.

We chose 4T1 and Bcap37 cell lines as the model cells for this study, because we were familiar with the nature of these cells: we have used these cells to study cancer cell survival under glucose deprivation [23], glycolytic flux control [24], glucose metabolism and cellular energetics [25, 26], ROS generation and cytosolic NAD/NADH ratios [27, 28], chromosomal instability under stress condition [29], intervention of metastasis by schisandrin B [30, 31].

## RESULTS

### Clones derived from 4T1 cells exhibit diversity of drug response pattern

**Figure 1:**
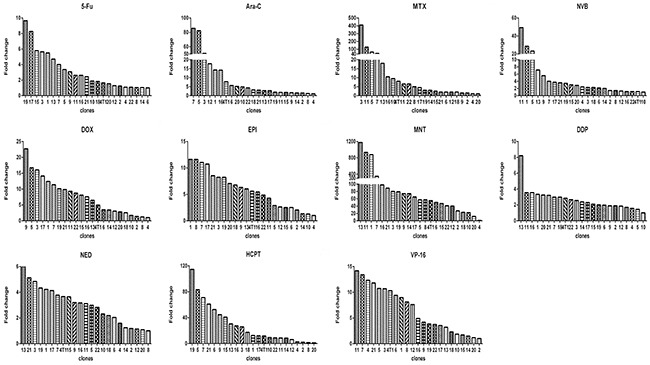
Relative drug sensitivity of clones from 4T1 cells For each drug, there was a clone that had the smallest IC_50_, which was used to divide IC_50_ of this clone and of all other clones (x axis) to derive the value of fold change (y axis).

**Figure 2:**
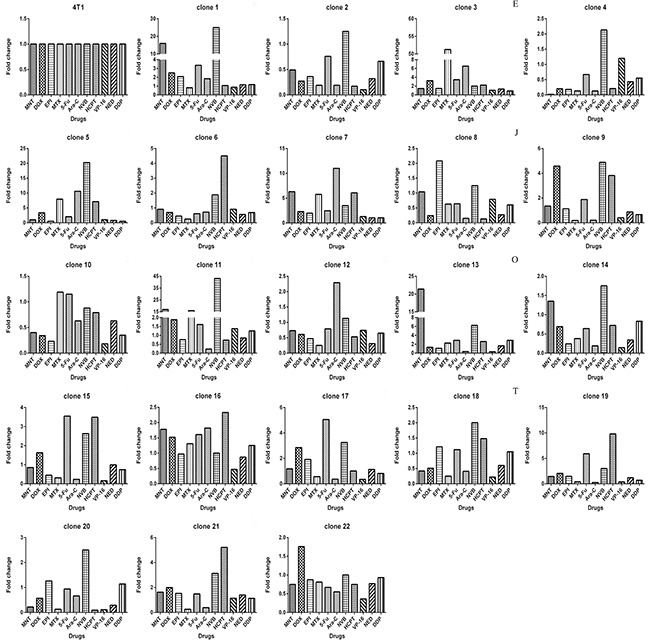
Each clone derived from 4T1 cells exhibits drug-response pattern distinct from others The IC_50_s of 4T1 cells toward 11 drugs (x axis) were used to divide IC_50_s of 4T1 cells and of all other clones to derive fold change values (y axis).

Using limited dilution culture, we obtained 22 single cell-derived clones from 4T1 cell line. These clones were expanded and assayed for their sensitivity to a panel of 11 anticancer drugs, including antimetabolites, anthracycline compounds, plants chemicals and platinum drugs (Table 1). Two prominent points were observed. First, sensitivities to each drug varied significantly from clone to clone, e.g., clone 13 was 1176 folds more resistant to MNT than clone 4, clone 19 was 115 folds more resistant to HCPT than clone 20, clone 3 was 410 folds more resistant to MTX than clone 20, among others (Figure 1). Second, each clone exhibited distinct drug-response pattern (Figure 2). In comparison to 4T1 cells, one clone could be more resistant to some drug but more sensitive to others, e.g., clone 13 was resistant to MNT but sensitive to Ara-C, clone 1 was resistant to MNT and NVB but sensitive to MTX, among others.

**Table 1:** IC_50_ (μg/ml) of clones from 4T1

Drugs/clones	MNT	DOX	EPI	MTX	5-Fu	Ara-C	NVB	HCPT	VP-16	Ned	DDP
1	0.877±0.018	0.558±0.089	0.395±0.021	0.013±0.001	0.555±0.064	1.03±0.113	0.2±0.028	0.547±0.202	5.145±0.774	3.04±0.226	0.885±0.064
2	0.027±0.008	0.061±0.013	0.069±0.004	0.003±0	0.125±0.007	0.105±0.064	0.01±0.003	0.087±0.021	0.574±0.18	0.85±0.156	0.5±0.014
3	0.08±0.006	0.721±0.171	0.28±0.099	0.82±0.428	0.57±0.071	3.65±0	0.016±0.001	1.134±0.141	6.136±1.359	3.485±0.247	0.68±0.057
4	0.001±0	0.045±0.006	0.034±0.011	0.002±0	0.11±0.014	0.072±0.011	0.017±0.007	0.107±0.009	7.081±1.394	1.145±0.445	0.415±0.064
5	0.057±0.002	0.751±0.004	0.098±0.032	0.127±0.1	0.34±0.141	5.935±0.417	0.162±0.097	3.662±0.542	6.169±0.581	2.15±0.311	0.38±0.057
6	0.05±0.006	0.156±0.053	0.085±0.021	0.004±0.001	0.101±0.013	0.405±0.205	0.015±0.001	2.312±0.478	5.396±1.684	1.47±0.156	0.52±0
7	0.347±0.031	0.511±0.015	0.375±0.007	0.092±0.017	0.405±0.078	6.165±0.658	0.028±0.003	3.12±0.026	7.688±1.401	2.72±0.141	0.79±0.028
8	0.057±0.004	0.054±0.003	0.395±0.049	0.01±0.001	0.105±0.008	0.085±0.021	0.01±0	0.066±0.025	4.652±0.102	0.72±0.24	0.45±0.028
9	0.074±0.001	1.021±0.345	0.215±0.163	0.003±0	0.31±0.099	0.115±0.078	0.039±0.012	1.965±0.933	2.398±0.262	2.305±0.332	0.5±0.141
10	0.022±0.001	0.076±0.006	0.043±0.005	0.019±0.001	0.19±0.085	0.35±0	0.007±0.001	0.405±0.06	1.069±0.169	1.665±0.021	0.265±0.049
11	0.933±0.029	0.419±0.025	0.145±0.021	0.255±0.095	0.265±0.007	0.13±0.014	0.345±0.092	0.377±0.021	8.143±0.607	2.245±0.219	0.94±0.014
12	0.04±0.021	0.137±0.011	0.089±0.016	0.004±0.001	0.13±0	1.28±0.028	0.009±0.001	0.272±0.024	4.376±0.509	0.82±0.085	0.49±0
13	1.176±0.092	0.291±0.118	0.205±0.007	0.036±0.016	0.475±0.035	0.2±0	0.05±0.028	1.334±0.214	1.833±0.263	4.32±0.141	2.175±0.12
14	0.074±0.011	0.153±0.008	0.045±0.026	0.006±0.001	0.105±0.007	0.107±0.018	0.014±0.001	0.369±0.068	0.846±0.092	0.895±0.064	0.63±0.311
15	0.047±0.025	0.363±0.14	0.085±0.008	0.005±0	0.585±0.134	0.13±0.014	0.021±0.005	1.792±0.066	0.974±0.513	2.625±0.233	0.555±0.021
16	0.098±0.018	0.34±0.038	0.185±0.021	0.021±0.008	0.265±0.064	1.02±0.665	0.008±0.004	1.199±0.022	2.813±0.486	2.285±0.064	0.94±0.099
17	0.065±0.013	0.635±0.047	0.365±0.035	0.009±0.001	0.835±0.247	0.2±0.014	0.026±0.006	0.52±0.028	2.027±0.35	2.975±0.544	0.61±0.042
18	0.023±0.002	0.114±0.006	0.23±0.127	0.004±0.001	0.185±0.021	0.23±0.057	0.016±0.005	0.762±0.187	1.32±0.448	1.575±0.841	0.79±0.113
19	0.079±0.015	0.454±0.185	0.28±0.071	0.006±0.001	0.975±0.233	0.14±0.014	0.024±0.004	5.044±3.003	2.199±0.708	3.12±0.481	0.53±0.028
20	0.012±0.001	0.126±0.002	0.24±0.042	0.002±0	0.155±0.007	0.37±0.028	0.02±0.008	0.044±0.012	0.664±0.095	0.775±0.134	0.86±0.127
21	0.089±0.006	0.443±0.199	0.29±0.127	0.004±0	0.245±0.106	0.22±0.028	0.025±0.003	2.68±0.877	6.776±0.939	3.69±0.17	0.85±0.057
22	0.041±0.001	0.392±0.023	0.165±0.007	0.013±0.001	0.11±0.015	0.31±0.042	0.008±0	0.385±0.133	2.15±0.077	2.035±0.573	0.705±0.049
Resistant clone	13	9	1,8	3	19	7	11	19	11	13	13
Sensitive clone	4	4	4	20	6	4	10	20	2	8	10
Ratio (resistant/sensitive)	1176	22.7	11.6	410	9.7	85.6	49.3	114.6	14.2	6	8.2

Cells were incubated with a series of different concentrations of drugs for 48 hours and subjected for a MTT assay to measure half inhibitory concentration (IC50). Data were means ± SD of two independent experiments.

**Figure 3:**
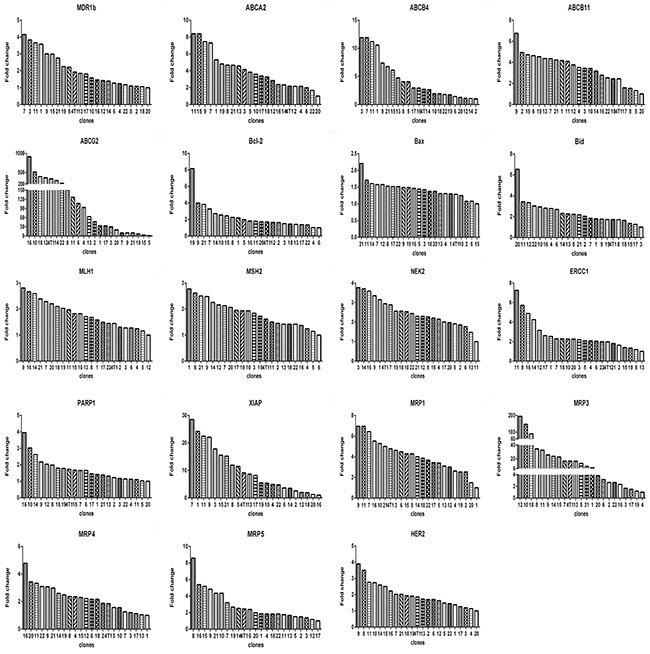
Relative levels of gene expression of clones from 4T1 cells For each gene, there was a clone that had the lowest expression level. The fold change is based on the formula 2^-[(ΔCT)clone(i)-(ΔCT)clone(a)]^ according to the method previously described [39], where, clone(i) denote any one of the 22 clones, and clone(a) denotes the one with lowest expression of a given gene. ΔCT was derived as described in Materials and Methods.

**Figure 4:**
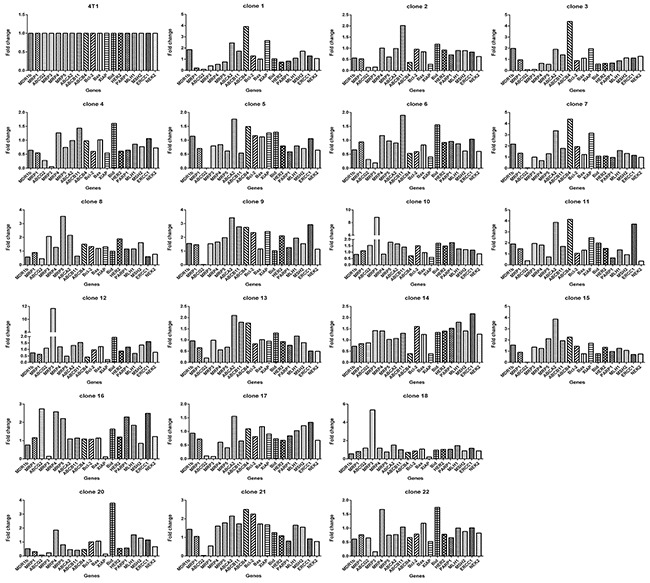
Each clone derived from 4T1 cells exhibits gene expression pattern distinct from others Taking 4T1 as a reference, The relative expression level of the 19 genes in each clone is based on the formula 2^-[(ΔCT)clone(i)-(ΔCT)4T1]^ according to the method previously described [39], where, clone(i) denote any one of the 22 clones. ΔCT was derived as described in Materials and Methods.

Next, we measured the expression of a panel of 19 genes associated with drug response, including ATP-binding cassette (ABC) transporter family, the mismatch repair (MMR) system, chromosomal instability genes, DNA damage repair genes and apoptosis related genes. Like their response to drugs, each clone exhibited distinct gene expression profiles (Figure 3 & 4).

### Daughter clones from a single 4T1 cell show heterogeneous phenotypes

**Figure 5:**
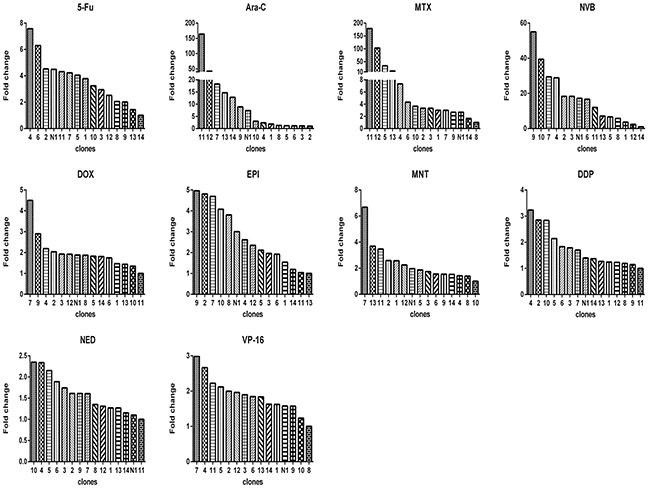
Relative drug sensitivity of subclones from monoclonal N1 (a clone from 4T1) For each drug, there was a clone that had the smallest IC_50_, which was used to divide IC_50_ of this subclone and of all other subclones (x axis) to derive the value of fold change (y axis).

**Figure 6:**
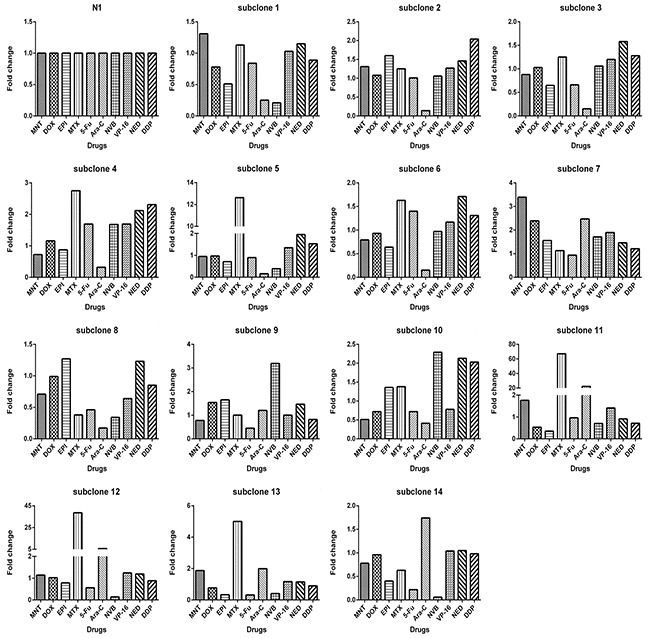
Each subclone derived from monoclonal N1 exhibits drug-response pattern distinct from others The IC_50_s of monoclonal N1 toward 10 drugs (x axis) were used to divide IC_50_s of the monoclonal N1 and of all other subclones to derive fold change values (y axis).

The above results indicated that clones from 4T1 cells were significantly different from each other. This seemed to suggest that each 4T1 cell was unique and such a high heterogeneity of 4T1 cells was beyond our expectation. We thought that the difference might be largely phenotypic. It would be expensive to investigate the question using single cell sequencing technology. The alternative approach is to check if clones derived from a single 4T1 cell were phenotypically different. Using limited dilution culture, we isolated a single cell-derived clone N1. This clone was then further subcloned and 14 daughter clones were obtained. Again, the sensitivities to anticancer drugs varied remarkably from clone to clone (Table 2, Figure 5), e.g., subclone 11 was 164 folds more resistant to Ara-C than subclone 2, subclone 9 was 55 folds more resistant to NVB than clone 14, subclone 4 was 7 folds more resistant to 5-Fu than subclone 14, among others. On the other hand, A subclone that was resistant to a drug could be sensitive to another, e.g., subclone 11 was resistant to MTX and Ara-C but sensitive to Dox and EPI. Each subclone exhibited distinct drug response profile from others (Figure 6).

**Table 2:** IC_50_ (μg/ml) of subclones of monoclonal N1

Drugs/Clones	MNT	DOX	EPI	MTX	5-Fu	Ara-C	NVB	VP-16	Ned	DDP
1	0.175±0.007	0.31±0.044	0.2±0	0.009±0.002	1.08±0.226	0.315±0.177	0.033±0.009	6.83±0.26	3.48±0.028	0.93±0.085
2	0.176±0.027	0.43±0.074	0.625±0.064	0.01±0.003	1.29±0.339	0.17±0.014	0.165±0.021	8.395±0.52	4.415±0.672	2.14±0.679
3	0.118±0.004	0.407±0.04	0.255±0.049	0.01±0	0.84±0.141	0.185±0.049	0.165±0.021	7.984±0.2	4.775±0.304	1.34±0.113
4	0.097±0.04	0.462±0.029	0.34±0	0.022±0.006	2.16±0.297	0.4±0.099	0.26±0.127	11.209±0.273	6.41±0.396	2.425±0.276
5	0.127±0.037	0.385±0.013	0.275±0.007	0.101±0.04	1.155±0.078	0.2±0.028	0.06±0.003	8.915±0.639	5.895±0.46	1.605±0.148
6	0.106±0.035	0.369±0.048	0.25±0.014	0.013±0.006	1.795±0.474	0.19±0.014	0.15±0.113	7.76±0.598	5.175±0.94	1.375±0.078
7	0.454±0.02	0.95±0.391	0.61±0.028	0.009±0.001	1.205±0.177	3.1±0.608	0.265±0.021	12.568±1.616	4.405±0.035	1.275±0.021
8	0.095±0.008	0.395±0.107	0.495±0.134	0.003±0	0.585±0.177	0.215±0.049	0.052±0.002	4.208±0.997	3.7±0.198	0.895±0.007
9	0.105±0.004	0.612±0.061	0.645±0.092	0.008±0.001	0.575±0.233	1.51±0.354	0.495±0.078	6.618±2.085	4.41±0.354	0.86±0.085
10	0.068±0.023	0.284±0.071	0.53±0.113	0.011±0.004	0.925±0.007	0.51±0.212	0.355±0.007	5.189±0.076	6.435±1.549	2.13±0.453
11	0.236±0.013	0.211±0.001	0.135±0.049	0.537±0.016	1.23±0.368	27.85±3.748	0.108±0.031	9.344±0.073	2.74±0.17	0.75±0.099
12	0.153±0.045	0.406±0.006	0.305±0.035	0.309±0.023	0.72±0.042	7.18±1.64	0.022±0.001	8.249±0.233	3.59±0.113	0.925±0.035
13	0.251±0.018	0.304±0.013	0.13±0.014	0.04±0.009	0.41±0.085	2.495±1.025	0.064±0.04	7.726±0.09	3.475±0.078	0.95±0
14	0.104±0.02	0.382±0.17	0.155±0.007	0.005±0.001	0.285±0.021	2.18±0.255	0.009±0.001	6.861±0.175	3.16±0.014	1.03±0.014
Resistant clone	7	7	9	11	4	11	9	7	10	4
Sensitive clone	10	11	3	8	14	2	14	8	11	11
Ratio (resistant/sensitive)	6.7	4.5	5.0	179	7.6	163.8	55	3.0	2.4	3.2

Cells were incubated with a series of different concentrations of drugs for 48 hours and subjected for a MTT assay to measure half inhibitory concentration (IC50). Data were means ± SD of two independent experiments.

**Figure 7:**
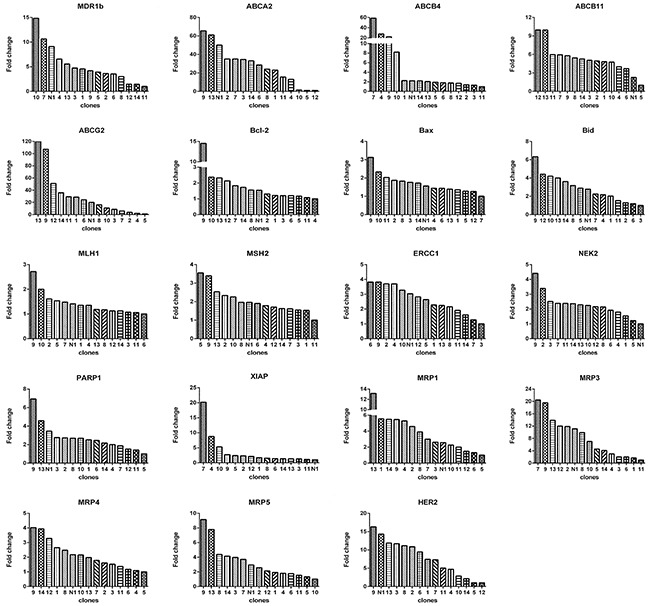
Relative levels of gene expression of subclones from monoclonal N1 For each gene, there was a subclone that had the lowest expression level. The fold change is based on the formula 2^-[(ΔCT)subclone(i)-(ΔCT)subclone(a)]^ according to the method previously described [39], where, subclone(i) denote any one of the 14 subclones, and subclone(a) denotes the one with lowest expression of a given gene. ΔCT was derived as described in Materials and Methods.

**Figure 8:**
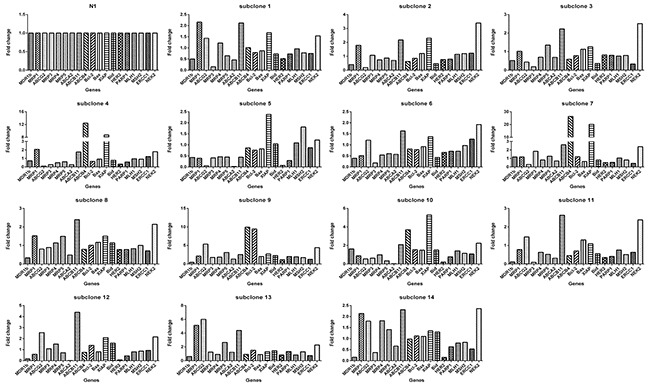
Each subclone derived from monoclonal N1 exhibits gene expression pattern distinct from others Taking N1 as a reference, the relative expression level of the 19 genes in each subclone is based on the formula 2^-[(ΔCT)subclone(i)-(ΔCT)N1]^ according to the method previously described [39], where, subclone(i) denote any one of the 14 clones. ΔCT was derived as described in Materials and Methods.

Then we determined the expression of 19 genes in these subclones. Again, we observed 2 prominent points: (1) the expression levels of genes among subclones could differ remarkably (Figure 7), e.g., subclone 10 expressed *MDR1b* by 15 folds higher than subclone 11, subclone 13 expressed *ABCG2* by 120 higher than subclone 5, among others; (2) Each subclone exhibited a distinct gene expression profile (Figure 8). A subclone that displayed relatively low level of some transcripts could expressed high levels of other transcripts, e.g., subclone 10, although had relatively lower levels of *ABCG2* and *MRP5* transcripts, showed the highest transcript level of *MDR1b*.

The results obtained from daughter clones derived from a single clone were interesting, but required repetition by independent experiment. We then isolated another single cell clone from 4T1, which was then subcloned and 12 daughter clones were obtained. Similar results regarding drug-response patterns and gene expression profiles were obtained (Supplementary Table 1, Supplementary Figure 1-4).

### Heterogeneous phenotypes of clones from Bcap37 cell line or from a single Bcap37 cell

Like clones derived from 4T1, the 10 single-cell derived clones from Bcap37 cell line also exhibited diversity in drug response patterns (Supplementary Table 2, Supplementary Figure 5-6). Unlike clones derived from 4T1, the difference of the fold change between clones in drug sensitivity was much smaller, e.g., the biggest fold change was about 8 (HCPT), whereas in 4T1 clones the biggest fold change was 1176 (MNT). It was noted that the absolute number of IC_50_s of Bcap37 and its clones were substantially higher than those of 4T1 and its clones, indicating the specific feature of individual cell lines.

The daughter clones from a single Bcap37 cell origin also exhibited a similar feature to those derived from Bcap37 cell line as described above (Supplementary Table 3): the drug sensitivity between subclones varied (Supplementary Figure 7) and each subclone exhibited distinct drug-response pattern (Supplementary Figure 8).

Next, we checked the expression of a panel of 18 drug resistance associated genes in the clones from the Bcap37 cell line or from a single Bcap37 cell. Like their response to drugs, each clone or subclone exhibited distinct gene expression profiles (Supplementary Figure 9-12).

## DISCUSSION

The main purpose of this study is to investigate how big the difference of daughter clones from a single cell origin can be. The key result of the study is the demonstration of significant difference of drug resistance/sensitivity between daughter clones from a single 4T1 cell origin. These daughter clones had the same genetic background, they were cultured under same condition, and they were not stimulated with anticancer drugs, yet they exhibited a remarkable diversity of drug response pattern. The fold change (drug sensitivity index) could be markedly different from each other, e.g., the most resistant daughter clone (clone 11, Table 2) was 164 folds higher than the least resistant one (clone 2) responding to Ara-C. Previously, there were reports regarding the difference of daughter cells responding to AZ138 (an antimitotic drug) and camptothecin [22, 32], but both studies focused on different fates of the daughter cells other than the points addressed in this study. It is noted that the same phenomenon was observed in clones derived from a single Bcap37 cell, but the fold change of drug response (Supplementary Figure 5) was not as dramatic as those for clones obtained from a single 4T1 cell. The similarity and difference between 4T1 and Bcap37 may reflect the specific feature of individual cell lines.

The quick diversification of daughter clones from a single cell under the same culture without drug pretreatment condition suggests the elusive epigenetics. The expression levels of genes between daughter clones differed dramatically, e.g., the fold change of gene *ABCG2* could be as large as 120 (Figure 7). Each daughter clone displayed a distinct pattern of gene expression. Apparently, a drug response pattern was associated with a complex gene expression, as indicated by the expression levels of a panel of 19 genes.

We showed that clones with different drug response patterns existed in a cell line, which was not novel, as many studies previously have shown that clones form a cell line can vary from each other in many ways, including drug resistance [11, 33, 34], metastasis [34]. Two relatively fresh points in this study were: (1) remarkable difference between clones from 4T1 cells, the fold change of drug resistance, e.g, to MNT, between clones (Figure 1), can be as large as 3 orders of magnitude, the fold change of gene expression, such as *ABCG2*, can be as large as 2 orders of magnitude (Figure 3); (2) each clone in the 22 clones seems unique regarding drug response (Figure 2) or gene expression (Figure 4). Such a highly heterogeneous nature observed in 4T1 cells may simply reflect phenotypic difference, because a single cell through simple division can quickly produce an array of daughter clones dramatically different from each other. We assume that each cell can produce 2 daughter cells, the daughter cells further produce daughter cells; each division may produce some fluctuations, and the fluctuation could add up.

Taken together, even a single cancer cell, through simple division, without exogenous stimuli, can quickly and randomly generate an array of daughter clones with diverse drug-response phenotypes. This might be an important way for cancer cells to acquire diversity of drug resistance, in addition to the classical intrinsic and acquired drug resistance. This observation unravels to some extent the elusive nature of cancer cells, which may potentially interfere with chemotherapy.

## MATERIALS AND METHODS

### Chemicals

RPMI 1640 and fetal calf serum, 0.25% trypsin were purchased from GIBCO-BRL (Grand Island, NY, USA);TRIzol^®^ reagent was purchased from Life Technologies, Inc. (Rockville, MD, USA); primer pairs were synthesized by Sangon Co. (Shanghai, People's Republic of China); iTaq™ Universal SYBR Green^®^ Supermix was purchased from Bio-Rad Laboratories, Inc.(Hercules, CA, USA).

### Cell culture

Mouse breast cancer cell line (4T1) and human breast cancer cell line (Bcap37) were from the Cell Bank of the Chinese Academy of Sciences (Shanghai, China) and cultured in RPMI-1640 supplemented with 10% fetal bovine serum (FBS) and antibiotics. All cells were grown in 37°C, 5% CO_2_ in a humidified atmosphere.

### Clones from 4T1 or Bcap37 cell lines

Using limited dilution method, 4T1 cells were seeded in the 96-well plate with average one cell per well. After 14 days, single cell clones were isolated and expanded in RPMI-1640 medium supplemented with 10% fetal bovine serum (FBS) and antibiotics. The single cell clones from Bcap37 cells were acquired through the same method as 4T1.

### Subclones from a single 4T1 or Bcap37 cell

Using limited dilution method, monoclonal 4T1 or Bcap37 cells described above were seeded in the 96-well plate with average one cell per well. 14 days later, subclones were isolated and expanded in RPMI-1640 medium supplemented with 10% fetal bovine serum (FBS) and antibiotics.

### MTT assay

Cytotoxicity was measured by MTT assay. Briefly, 4T1 cells were trypsinized and seeded into 96-well plates at a density of 4×10^3^/well and Bcap37 cells were seeded at a density of 8×10^3^/well overnight. Then a series of different concentrations of drugs were added to the cells. After 48 hours incubation, MTT (Sigma-Aldrich) was added with a work concentration of 0.5 mg/ml. 4 hours later, 100 μl triplex solution (10% sodium dodecyl sulfate (SDS), 5% isobutanol,12 mmol/L HCl) was added to each well and continued to incubated in 37°C overnight. The plates were measured at 570nm and a reference wavelength of 630nm with a Bio-Rad model 680 microplate reader (Hercules, CA, USA). The percentage of cell survival was calculated by the following formula: percentage of cell survival = (mean absorbance in test wells)/(mean absorbance in control wells)×100%. Half inhibitory concentration (IC_50_) was determined [35].

### Real-time polymerase chain reaction

4T1 cells, Bcap37 cells, or monoclonal cells (clones or subclones) were collected and RNA was prepared using the TRIzol^®^ reagent according to the manufacturer's instruction [36]. The first strand cDNA was synthesized from extracted RNA using an Oligo dT as primer. Real time PCR reaction was carried out in 20 μl system containing the cDNA, primers, iTaq™ Universal SYBR Green^®^ Supermix, DEPC H_2_O and performed in a StepOne Plus™ machine (Applied Biosystems, Foster City, CA, USA). Primers sequences are listed in Supplementary table 4&5. A thermal profile of 30 second at 95 °C, followed by 40 cycles of 15 seconds at 95 °C and 60 seconds at 60 °C with real-time fluorescence measured at the end of each annealing step was used. Melting curve analysis was performed on each run to confirm a single peak of activity for each primer pair [37]. The fluorescent signal was determined using StepOne Plus™ software (Applied Biosystems, Foster City, CA, USA), giving the threshold cycle number (CT) at which PCR amplification reached a significant threshold. The ΔCT value is defined as the difference in CT value for the genes and GAPDH mRNA, the internal standard [38, 39].

The funders have no role in study design, in the collection, analysis, and interpretation of data, in the writing of the report, and in the decision to submit the paper for publication.

## SUPPLEMENTARY MATERIALS FIGURES AND TABLES




