# Supplementary material for: Evidence of drug-response heterogeneity rapidly generated from a single cancer cell
Source: Oncotarget. 2017 Apr 12;8(25):41113–24. doi: 10.18632/oncotarget.17064 (PMC5522224; doi:10.18632/oncotarget.17064)
Supplement: Supplementary file 1 [file oncotarget-08-41113-s001.pdf]

Evidence of drug-response heterogeneity rapidly generated from a single cancer cell

Supplementary Materials

SUPPLEMENTARY FIGURES AND TABLES

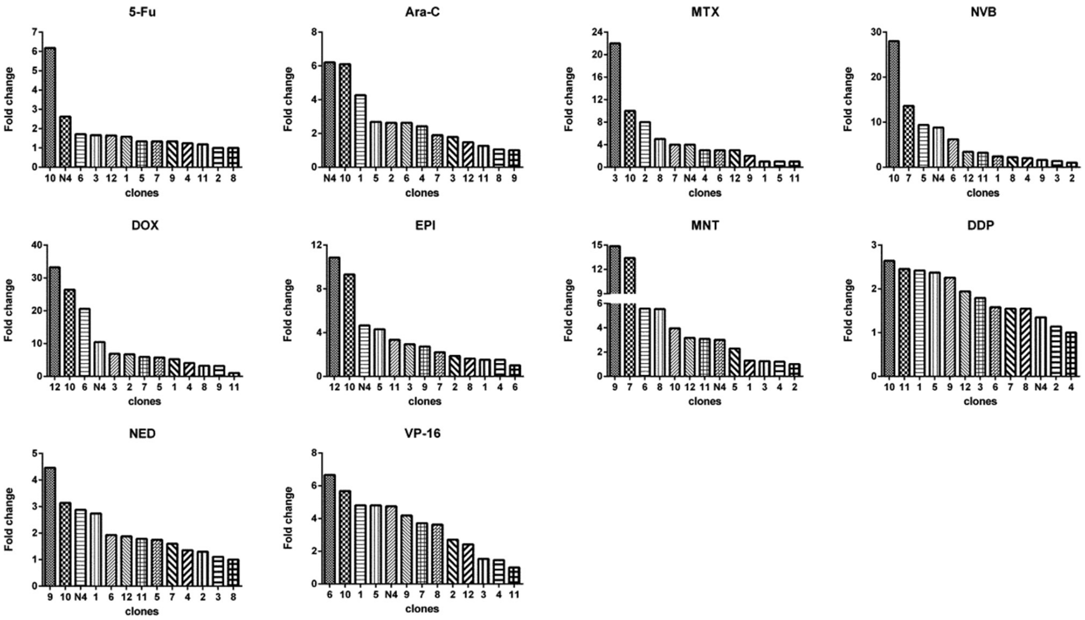

Supplementary Figure 1: Relative drug sensitivity of subclones from monoclonal N4 (a clone from 4T1). For each drug, there was a subclone that had the smallest IC_{50}, which was used to divide IC_{50} of this subclone and of all other subclones (x axis) to derive the value of fold change (y axis).

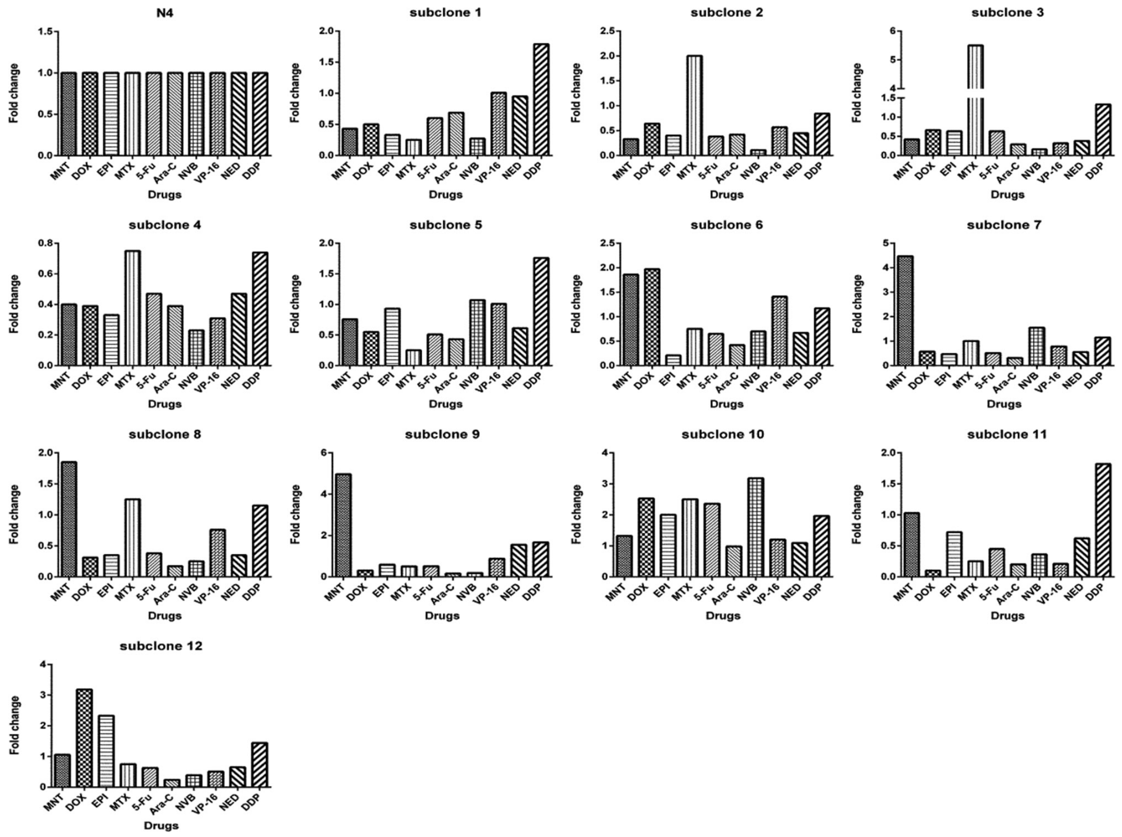

Supplementary Figure 2: Each subclone derived from monoclonal N4 exhibits drug-response pattern distinct from others. The IC_{50}s of monoclonal N4 toward 10 drugs (x axis) were used to divide IC_{50}s of the monoclonal N4 and of all other subclones to derive fold change values (y axis).

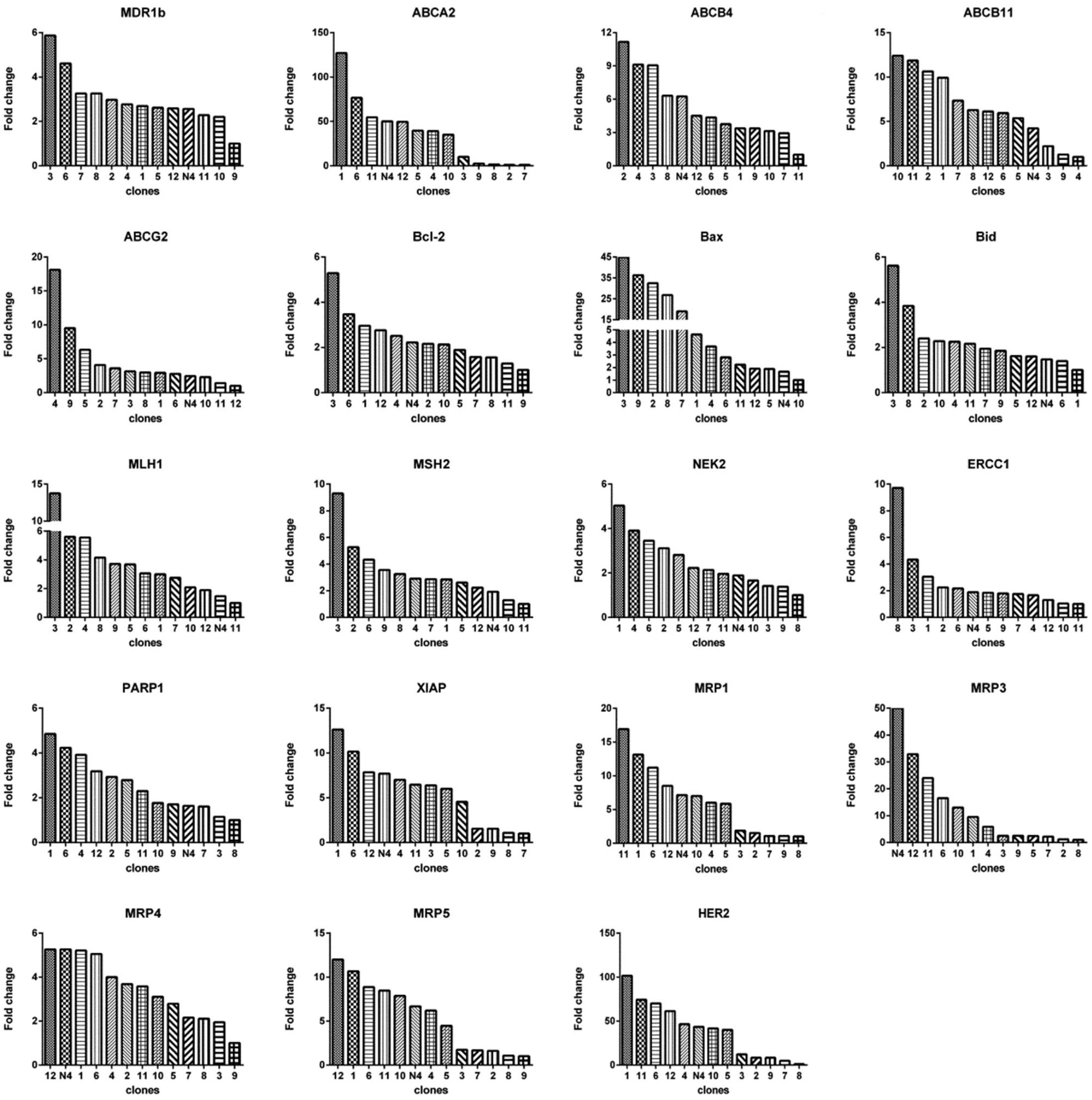

Supplementary Figure 3: Relative levels of gene expression of subclones from monoclonal N4. For each gene, there was a subclone that had the lowest expression level. The fold change is based on the formula $2^{-[(\Delta CT)subclone(i)-(\Delta CT)subclone(a)]}$ according to previously described, where, subclone(i) denote any one of the 12 subclones, and subclone(a) denotes the one with lowest expression of a given gene. ΔCT was derived as described in Materials and Methods.

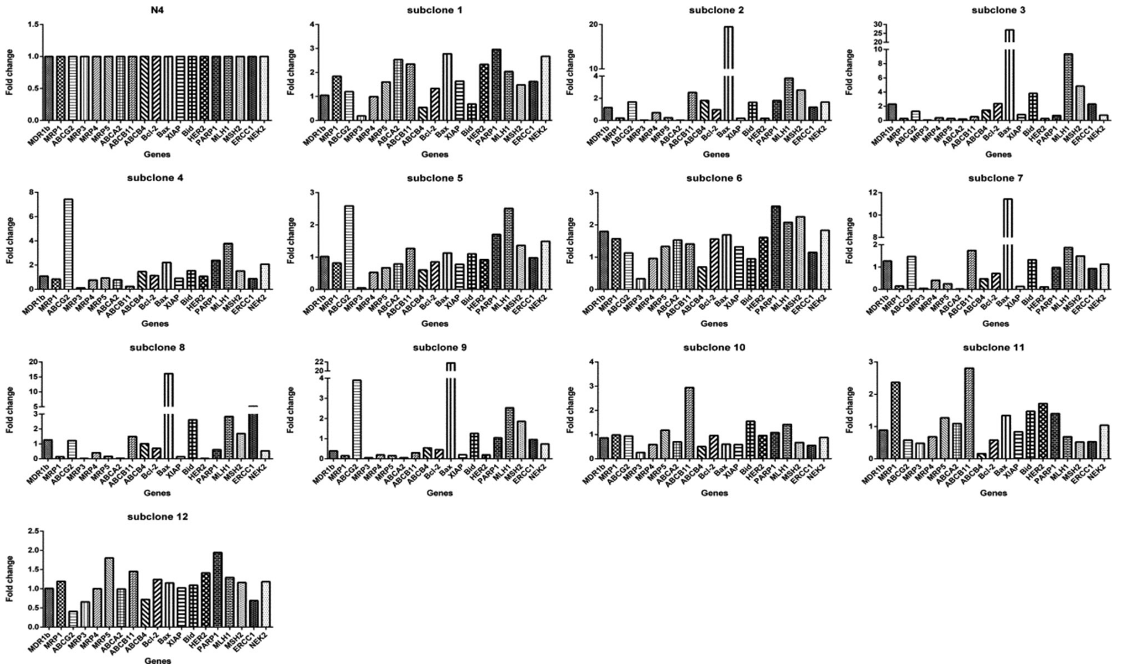

Supplementary Figure 4: Each subclone derived from monoclonal N4 exhibits gene expression pattern distinct from others. Taking N4 as a reference, the relative expression level of the 19 genes in each subclone is based on the formula $2^{-[(\Delta CT)subclone(i)-(\Delta CT)N4]}$ according to the method previously described, where, subclone(i) denote any one of the 12 clones. ΔCT was derived as described in Materials and Methods.

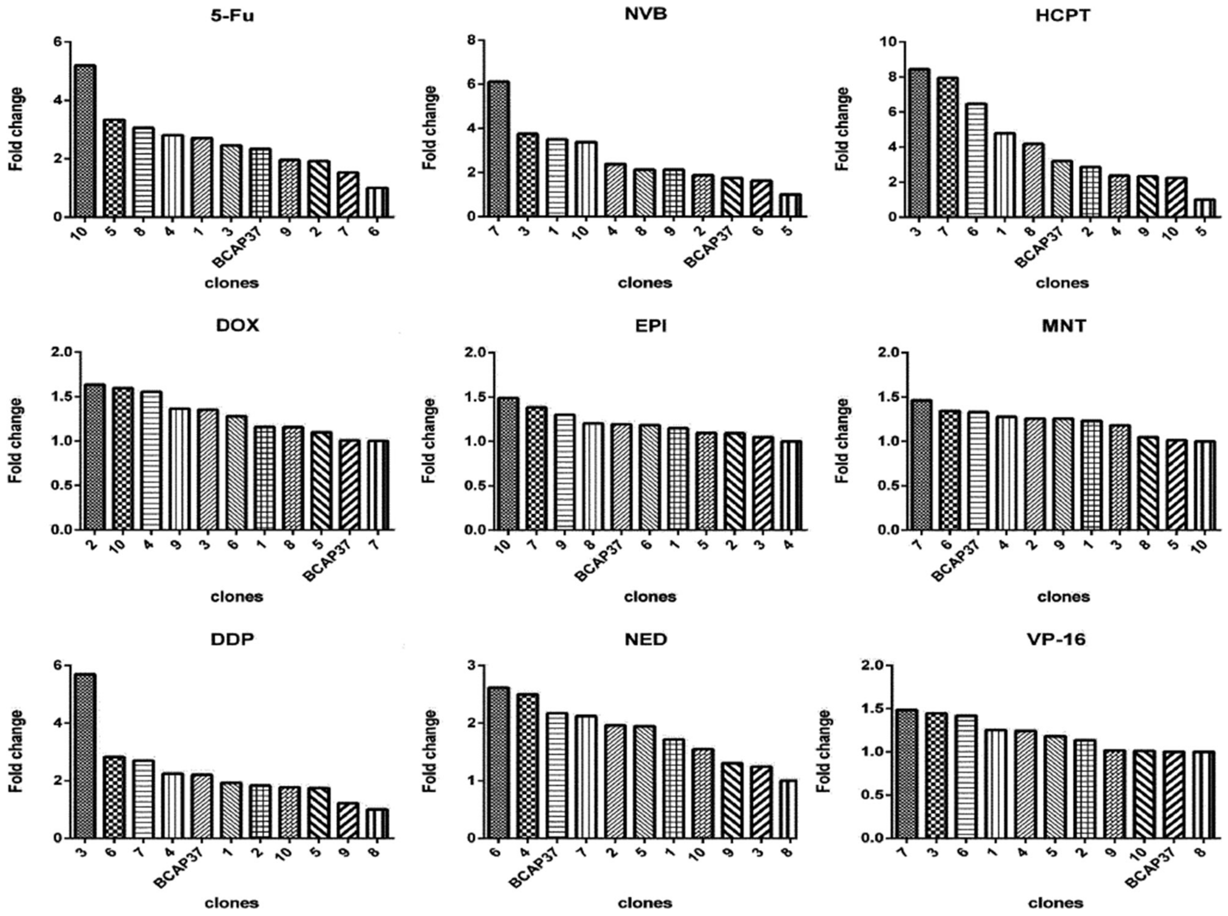

Supplementary Figure 5: Relative drug sensitivity of clones from Bcap37 cells. For each drug, there was a clone that had the smallest IC_{50}, which was used to divide IC_{50} of this clone and of all other clones (x axis) to derive the value of fold change (y axis).

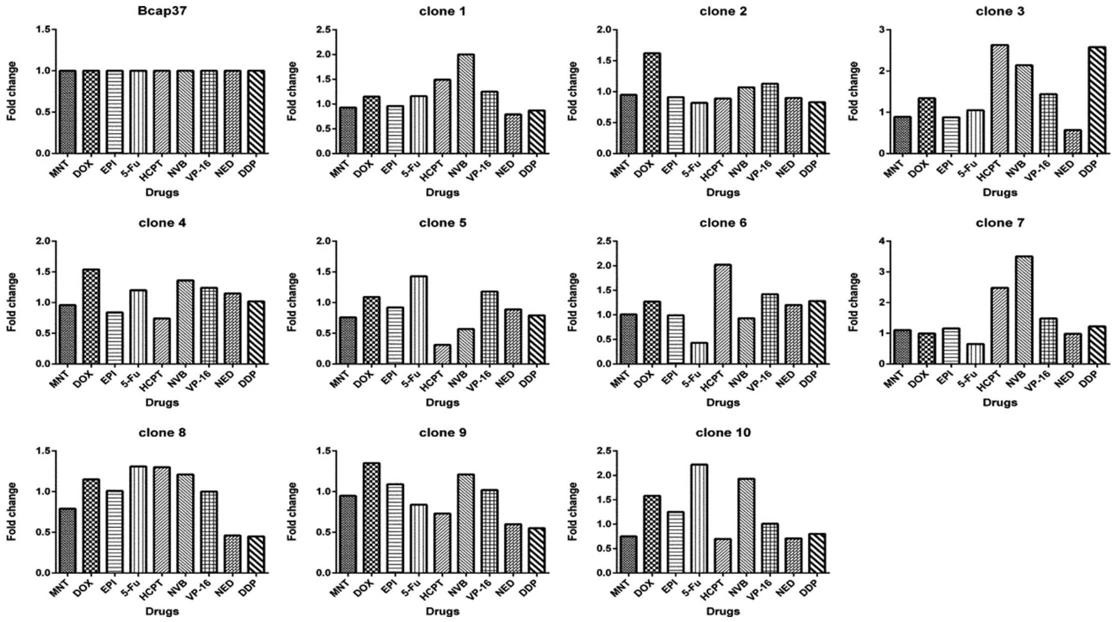

Supplementary Figure 6: Each clone derived from Bcap37 cells exhibits drug-response pattern distinct from others. The IC_{50}s of Bcap37 cells toward 9 drugs (x axis) were used to divide IC_{50}s of Bcap37 cells and of all other clones to derive fold change values (y axis).

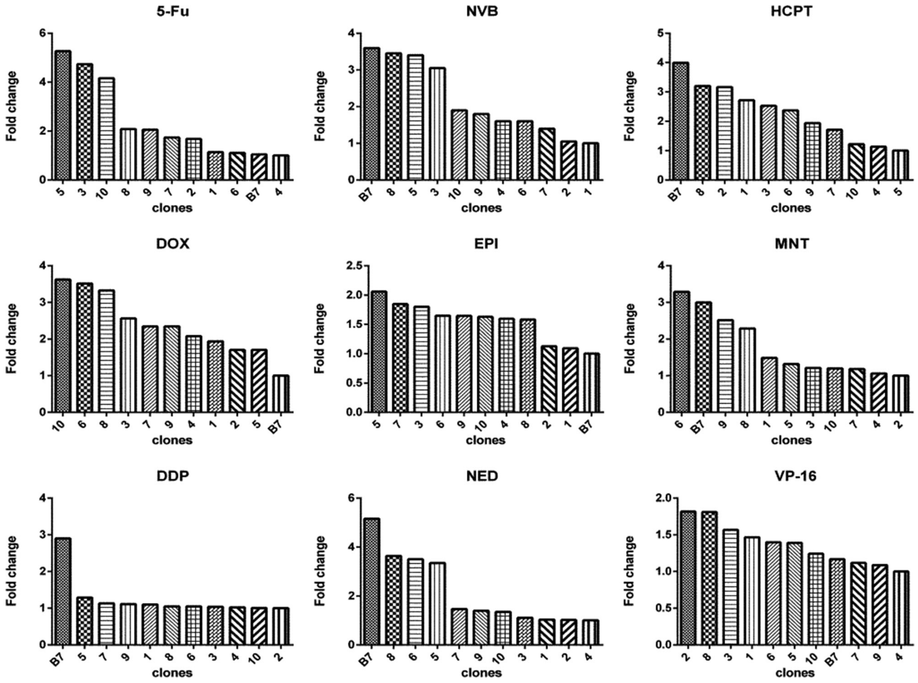

Supplementary Figure 7: Relative drug sensitivity of subclones from monoclonal B7 (a clone from Bcap37). For each drug, there was a clone that had the smallest IC_{50}, which was used to divide IC_{50} of this subclone and of all other subclones (x axis) to derive the value of fold change (y axis).

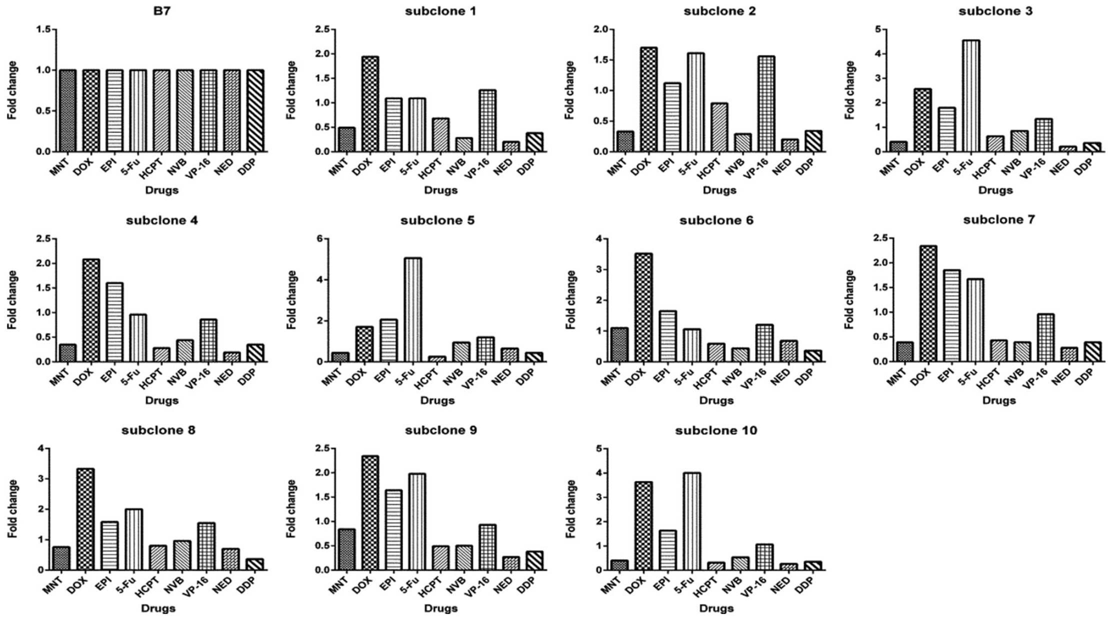

Supplementary Figure 8: Each subclone derived from monoclonal B7 exhibits drug-response pattern distinct from others. The IC_{50}s of monoclonal B7 toward 9 drugs (x axis) were used to divide IC_{50}s of the monoclonal B7 and of all other subclones to derive fold change values (y axis).

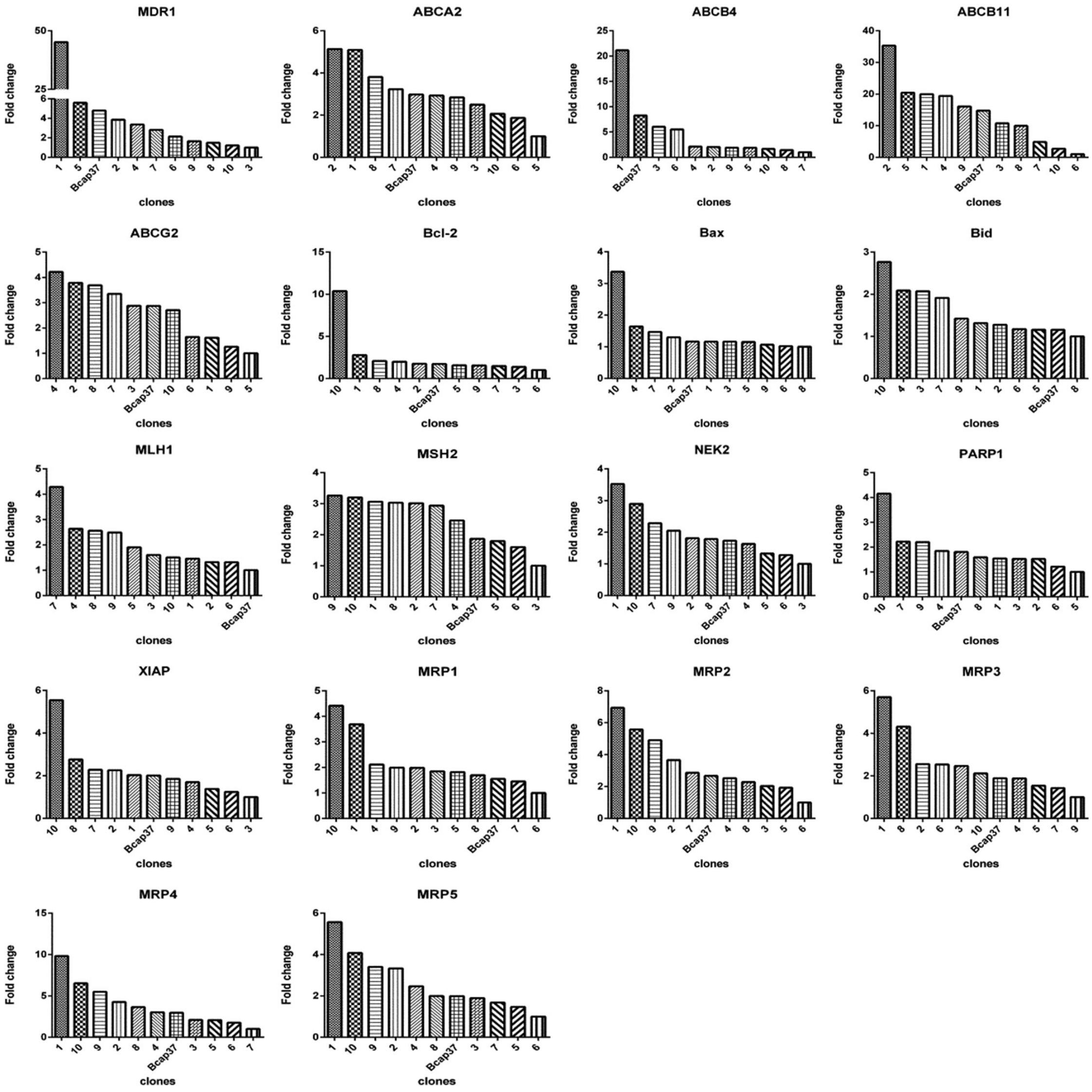

Supplementary Figure 9: Relative levels of gene expression of clones from Bcap37 cells. For each gene, there was a clone that had the lowest expression level. The fold change is based on the formula $2^{-[(\Delta CT)clone(i)-(\Delta CT)clone(a)]}$ according to previously described, where, clone(i) denote any one of the 10 clones, and clone(a) denotes the one with lowest expression of a given gene. ΔCT was derived as described in Materials and Methods.

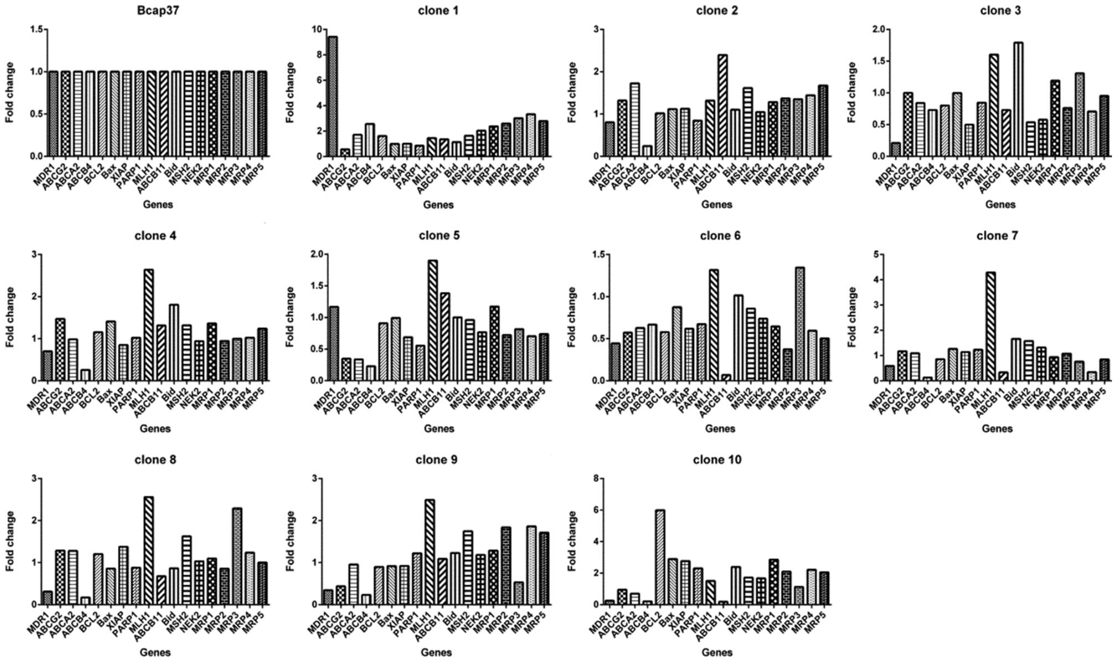

Supplementary Figure 10: Each clone derived from Bcap37 cells exhibits gene expression pattern distinct from others. Taking Bcap37 as a reference, the relative expression level of the 18 genes in each clone is based on the formula $2^{-[(\Delta CT)clone(i)-(\Delta CT)Bcap37]}$ according to the method previously described, where, clone(i) denote any one of the 10 clones. ΔCT was derived as described in Materials and Methods.

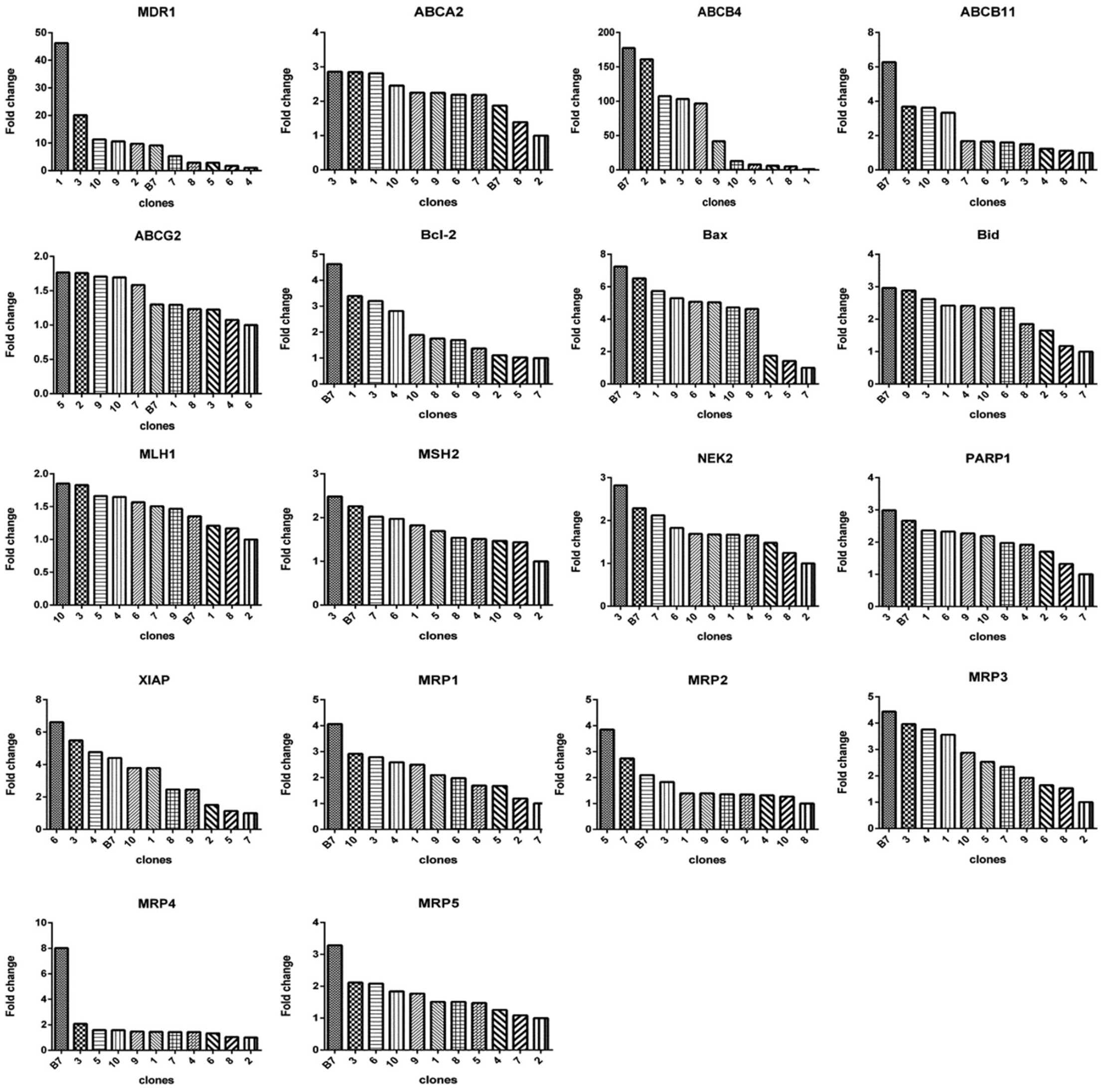

Supplementary Figure 11: Relative levels of gene expression of subclones from monoclonal B7. For each gene, there was a subclone that had the lowest expression level. The fold change is based on the formula $2^{-[(\Delta CT)subclone(i)-(\Delta CT)subclone(a)]}$ according to the method previously described [39], where, subclone(i) denote any one of the 10 subclones, and subclone(a) denotes the one with lowest expression of a given gene. ΔCT was derived as described in Materials and Methods.

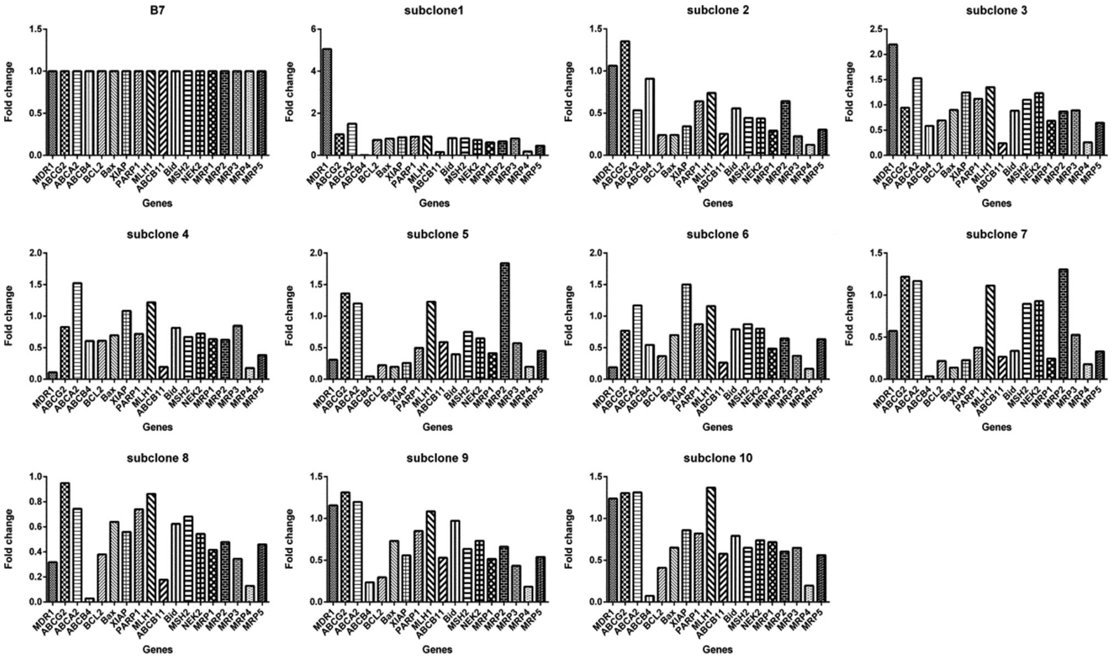

Supplementary Figure 12: Each subclone derived from monoclonal B7 exhibits gene expression pattern distinct from others. Taking B7 as a reference, the relative expression level of the 18 genes in each subclone is based on the formula $2^{-[(\Delta CT)subclone(i)-(\Delta CT)B7]}$ according to the method previously described [39], where, subclone(i) denote any one of the 10 subclones. ΔCT was derived as described in Materials and Methods.

Supplementary Table 1: IC_{50} (µg/ml) of subclones of monoclonal N4 (a clone from 4T1). Cells were incubated with a series of different concentrations of drugs for 48 hours and subjected for a MTT assay to measure half inhibitory concentration (IC_{50}). Data were means ± SD of two independent experiments.

See Supplementary File 1

Supplementary Table 2: IC_{50} (µg/ml) of single cell clones from Bcap37. Cells were incubated with a series of different concentrations of drugs for 48 hours and subjected for a MTT assay to measure half inhibitory concentration (IC_{50}). Data were means ± SD of two independent experiments.

See Supplementary File 1

Supplementary Table 3: IC_{50} (µg/ml) of subclones of monoclonal B7. Cells were incubated with a series of different concentrations of drugs for 48 hours and subjected for a MTT assay to measure half inhibitory concentration (IC_{50}). Data were means ± SD of two independent experiments.

See Supplementary File 1

Supplementary Table 4: Primer sequences of 4T1 cells and their clones or subclones

Gene	GenBank-EMBL number	Primer 1 (forward)	Primer 2 (reverse)
MDR1b	NM_011075.2	AGAAGCGAAGTTCAGATC	CCTCAGATACCTCACATTG
MRP1	NM_008576.3	AGTTGGATGTGAATGAGGAG	CACGAAGTTGATAATCAATTCC
ABCG2	NM_011920.3	GCTGTTCAGGTTATGTGGTTCAAG	AGTTCCGACCTTAGAATCTGCTAC
MRP3	NM_029600.3	GCAATATACTTCCTCTGGCAGATC	TGATGCGGGAGTCCTTGAAC
MRP4	NM_001163676.1	TTCAGGACTGGTGGCTTTC	GGTGACCGCAGTTAGACC
MRP5	NM_013790.2	CTTCCATCTGACTGCGGCACAGG	ACAGCCACTGATGCTTCAGAGAGG
ABCA2	NM_007379.2	CCAGTGGAAGGAGATGAC	GCAGCCCATACATACCTG
ABCB11	NM_021022.3	ACATCCGCCTTGGTAGAGAAGAG	TCTCCAACTAGGGTGTCAAATTGC
ABCB4	NM_008830.2	AGGCCGCACTGGATAAGG	TCTGGCTTCCTGCTGTCTG
Bcl-2	NM_177410.3	GGATAACGGAGGCTGGGATG	CAGGCTGAGCAGGGTCTTC
Bax	NM_007527.3	GAGGCAGCGGCAGTGATG	TGCTCGATCCTGGATGAAACC
XIAP	NM_001301639.1	CCCAAATTCAACAAACTCTCCAAG	ACTTCACTTTATCGCCTTCACC
Bid	NM_007544.3	CCAAGGAAGAATAGAGCCAGATTC	TCCGACAGGCTGCCATTC
PARP1	NM_007415.2	CCTGGAGAAGATAGAGAAGG	CTGACTGGCACTGTACTC
MLH1	NM_026810.2	GGTGTTCTGAGGTTATCGG	CTCTGTCCAGCCACTTTC
MSH2	NM_008628.2	GGCGTGAAGTTTACCAACAGTG	GCTAACAATGGCGTCTAAGTGAG
ERCC1	NM_001127324.1	TGGGAAGGACGAGGAAAG	ATCTGAATAAGGGCTTGACC
NEK2	NM_010892.3	GGCTGCCTGCTGTATGAG	CTGAAGGTCGATGGTAGTCC
HER2	NM_001003817.1	GATGTCCTCCGTAAGAATAAC	CAGATGGTGCCAGTCAAG
GAPDH	NM_001289726.1	TGTTTGTGATGGGTGTGAAC	ATGGCATGGACTGTGGTC

Supplementary Table 5: Primer sequences of Bcap37 cells and their clones or subclones

Gene	GenBank-EMBL number	Primer 1 (forward)	Primer 2 (reverse)
MDR1	NM_000927.4	TTGGGGCTTTTAGTGTTG	GCTGTCAATACTTGGCTTA
ABCG2	NM_001257386.1	GCAACAACTATGACGAATC	AACTGAGTTCCAACCTTG
MRP1	NM_004996.3	TTACTCATTCAGCTCGTCTTG	ACTCTGGGCAGGGATTAG
MRP2	NM_000392.4	CTCCTACGGATTCCAGAT	TGTAGCCTTTCAGAATGATG
MRP3	NM_001144070.1	TACTCTCTGCCCTCATCT	TCTCAGGGTAGGGGTTAG
MRP4	NM_001105515.2	AGTTGTTCTCATCACTGA	ATCTTGGAAATCTCCTTCT
MRP5	NM_001023587.2	CGGACTACTTCCAAACAC	GAGAAGAAAGCCACGAAA
ABCA2	NM_001606.4	TGGACAGATCCACAGTGT	CAAGTTTTGCGTCAGGAAA
ABCB4	NM_000443.3	CTTCTCCTTTCCAGTGAA	CAATCCTGAGTAGTAATATGC
ABCB11	NM_003742.2	CAGACCAGAGGTGAAGAT	ATAGAATCGCTGAATGAGTT
Bcl-2	NM_000633.2	CGACTCCTGATTCATTGG	TCTACTTCCTCTGTGATGT
Bax	NM_001291428.1	CAAACTGGTGCTCAAGGC	CGGAGGAAGTCCAATGTC
XIAP	NM_001167.3	CTTGAGGAGTGTCTGGTA	CCATTCGTATAGCTTCTTGTA
Bid	NM_001196.3	CTTGCTCCGTGATGTCTT	CGTTCAGTCCATCCCATT
PARP1	NM_001618.3	AAAGAAAGTGTGTTCAACTAATG	GCTACTCGGTCCAAGATC
MLH1	NM_000249.3	ATCTGGATATTGTATGTGAA	ATCTGAGTAACTTGCTCT
MSH2	NM_000251.2	ATCAGTTCTCCAATCTTG	TCCTCCTCTTTGAATTATC
NEK2	NM_001204182.1	AACATCGTTCGTTACTAT	CTCTTCATCTAAGTATTGC
GAPDH	NM_001256799.2	TTGACCTCAACTACATGG	AAGATGGTGATGGGATTT
