# Supplementary material for: Evidence of drug-response heterogeneity rapidly generated from a single cancer cell
Source: Oncotarget. 2017 Apr 12;8(25):41113–24. doi: 10.18632/oncotarget.17064 (PMC5522224; doi:10.18632/oncotarget.17064)
Supplement: Supplementary file 2 [file oncotarget-08-41113-s002.docx]

Supplementary Table 1: IC_50_ (µg/ml) of subclones of monoclonal N4 (a clone from 4T1).Cells were incubated with a series of different concentrations of drugs for 48 hours and subjected for a MTT assay to measure half inhibitory concentration (IC_50_). Data were means ± SD of two independent experiments.

| Drugs  Clones | MNT | DOX | EPI | MTX | 5-Fu | Ara-C | NVB | VP-16 | NED | DDP |
| --- | --- | --- | --- | --- | --- | --- | --- | --- | --- | --- |
| 1 | 0.031±0.01 | 0.125±0.006 | 0.044±0.021 | 0.001±0 | 0.395±0.021 | 0.405±0.078 | 0.012±0.001 | 10.066±0.569 | 3.605±1.633 | 1.04±0.156 |
| 2 | 0.024±0.008 | 0.161±0.011 | 0.054±0.008 | 0.008±0 | 0.25±0.014 | 0.25±0.042 | 0.005±0 | 5.67±0.509 | 1.705±0.346 | 0.49±0.014 |
| 3 | 0.03±0.004 | 0.165±0.042 | 0.085±0.022 | 0.022±0.002 | 0.415±0.035 | 0.17±0.028 | 0.007±0 | 3.206±0.014 | 1.455±0.53 | 0.77±0.085 |
| 4 | 0.029±0.008 | 0.097±0.031 | 0.044±0.002 | 0.003±0.001 | 0.31±0.014 | 0.23±0 | 0.01±0.002 | 3.049±0.499 | 1.78±0.071 | 0.43±0 |
| 5 | 0.055±0.001 | 0.138±0.004 | 0.125±0.035 | 0.001±0 | 0.335±0.035 | 0.255±0.049 | 0.047±0.009 | 10.063±0.817 | 2.3±0.651 | 1.02±0.156 |
| 6 | 0.134±0.04 | 0.494±0.026 | 0.029±0.01 | 0.003±0 | 0.428±0.004 | 0.25±0.028 | 0.031±0.012 | 13.962±0.17 | 2.535±0.148 | 0.68±0.226 |
| 7 | 0.322±0.004 | 0.142±0.019 | 0.064±0.001 | 0.004±0 | 0.335±0.035 | 0.18±0.014 | 0.068±0.001 | 7.776±0.061 | 2.1±0.156 | 0.665±0.035 |
| 8 | 0.133±0.028 | 0.077±0.005 | 0.047±0.002 | 0.005±0 | 0.25±0.028 | 0.1±0.014 | 0.011±0 | 7.591±0.788 | 1.315±0.12 | 0.665±0.021 |
| 9 | 0.357±0.02 | 0.076±0.001 | 0.079±0.001 | 0.002±0 | 0.335±0.035 | 0.095±0.035 | 0.008±0 | 8.764±0.28 | 5.87±0.849 | 0.97±0.028 |
| 10 | 0.095±0.04 | 0.634±0.081 | 0.27±0.113 | 0.01±0.004 | 1.545±0.12 | 0.58±0.198 | 0.14±0.014 | 11.896±0.12 | 4.13±0.509 | 1.135±0.177 |
| 11 | 0.074±0.002 | 0.024±0.004 | 0.097±0.018 | 0.001±0 | 0.295±0.007 | 0.12±0.014 | 0.016±0.011 | 2.095±0.345 | 2.35±0.028 | 1.055±0.573 |
| 12 | 0.076±0.025 | 0.798±0.118 | 0.315±0.177 | 0.003±0 | 0.41±0.028 | 0.14±0.014 | 0.017±0.004 | 5.064±0.416 | 2.465±0.148 | 0.835±0.021 |
|  |  |  |  |  |  |  |  |  |  |  |
| Resistant clone | 9 | 12 | 12 | 3 | 10 | 10 | 10 | 6 | 9 | 10 |
| Sensitive clone | 2 | 11 | 6 | 1，5，11 | 2，8 | 9 | 2 | 11 | 8 | 4 |
| Ratio  (resistant/sensitive) | 14.9 | 33.3 | 10.9 | 22 | 6.2 | 6.1 | 28 | 6.7 | 4.5 | 2.6 |

Supplementary Table 2: IC_50_ (µg/ml) of single cell clones from Bcap37. Cells were incubated with a series of different concentrations of drugs for 48 hours and subjected for a MTT assay to measure half inhibitory concentration (IC_50_). Data were means ± SD of two independent experiments.

| drugs  clones | MNT | DOX | NED | DDP | EPI | NVB | VP-16 | HCPT | 5-Fu |
| --- | --- | --- | --- | --- | --- | --- | --- | --- | --- |
| 1 | 0.259±0.041 | 0.561±0.033 | 2.325±0.411 | 0.634±0.006 | 0.361±0.007 | 0.028±0.005 | 30.445±0.219 | 3.59±0.099 | 37±6.972 |
| 2 | 0.264±0.004 | 0.791±0.15 | 2.656±0.25 | 0.603±0.066 | 0.343±0.02 | 0.015±0.001 | 27.58±1.527 | 2.145±0.007 | 26.288±8.482 |
| 3 | 0.248±0.01 | 0.655±0.032 | 1.685±0.164 | 1.878±0.057 | 0.329±0.016 | 0.03±0.004 | 35.1±0.566 | 6.332±0.278 | 33.568±3.932 |
| 4 | 0.268±0.001 | 0.753±0.043 | 3.383±0.085 | 0.739±0.008 | 0.314±0.028 | 0.019±0.002 | 30.2±1.838 | 1.775±0.035 | 38.36±0.527 |
| 5 | 0.213±0.021 | 0.531±0.06 | 2.631±0.245 | 0.575±0.028 | 0.344±0.011 | 0.008±0.001 | 28.69±2.135 | 0.75±0.042 | 45.571±4.381 |
| 6 | 0.282±0.019 | 0.619±0.041 | 3.539±0.074 | 0.931±0.01 | 0.372±0.052 | 0.013±0.004 | 34.43±0.933 | 4.85±0.057 | 13.657±1.603 |
| 7 | 0.307±0.002 | 0.484±0.071 | 2.873±0.443 | 0.89±0.021 | 0.435±0.062 | 0.049±0.004 | 36.05±2.079 | 5.965±1.803 | 20.846±0.644 |
| 8 | 0.22±0.003 | 0.56±0.008 | 1.354±0.071 | 0.33±0.018 | 0.378±0.011 | 0.017±0.004 | 24.29±2.263 | 3.135±0.403 | 41.747±9.804 |
| 9 | 0.264±0.011 | 0.66±0.009 | 1.762±0.254 | 0.4±0.031 | 0.408±0.045 | 0.017±0.003 | 24.685±2.567 | 1.755±0.035 | 26.76±5.515 |
| 10 | 0.21±0.055 | 0.772±0.014 | 2.099±0.291 | 0.584±0.011 | 0.468±0.018 | 0.027±0.008 | 24.56±0.622 | 1.685±0.163 | 70.985±11.205 |
|  |  |  |  |  |  |  |  |  |  |
| Resistant clone | 7 | 2 | 6 | 3 | 10 | 7 | 7 | 3 | 10 |
| Sensitive clone | 10 | 7 | 8 | 8 | 4 | 5 | 8 | 5 | 6 |
| Ratio（resistant / sensitive） | 1.5 | 1.6 | 2.6 | 5.7 | 1.5 | 6.1 | 1.5 | 8.4 | 5.2 |

Supplementary Table 3: IC_50_ (µg/ml) of subclones of monoclonal B7. Cells were incubated with a series of different concentrations of drugs for 48 hours and subjected for a MTT assay to measure half inhibitory concentration (IC_50_). Data were means ± SD of two independent experiments.

| Drugs  clones | MNT | DOX | NED | DDP | EPI | NVB | VP-16 | HCPT | 5-Fu |
| --- | --- | --- | --- | --- | --- | --- | --- | --- | --- |
| 1 | 0.49±0.042 | 0.62±0 | 0.615±0.007 | 0.397±0.021 | 0.351±0.009 | 0.02±0.006 | 30.426±0.096 | 2.785±0.714 | 28.57±1.612 |
| 2 | 0.33±0.014 | 0.545±0.12 | 0.605±0.092 | 0.362±0.024 | 0.361±0.026 | 0.021±0.003 | 37.666±11.802 | 3.245±0.389 | 42.145±2.1 |
| 3 | 0.4±0.085 | 0.82±0.396 | 0.66±0.113 | 0.376±0.016 | 0.578±0.305 | 0.061±0.007 | 32.488±0.542 | 2.59±0.537 | 118.885±2.072 |
| 4 | 0.35±0.057 | 0.665±0.092 | 0.595±0.021 | 0.37±0.011 | 0.512±0.046 | 0.032±0.011 | 20.72±0.74 | 1.165±0.078 | 25.103±2.652 |
| 5 | 0.435±0.035 | 0.545±0.049 | 1.99±0.014 | 0.466±0.009 | 0.661±0.011 | 0.068±0.013 | 28.794±7.441 | 1.025±0.134 | 132.297±24.188 |
| 6 | 1.085±0.12 | 1.125±0.474 | 2.08±0.212 | 0.379±0.028 | 0.529±0.037 | 0.032±0.006 | 28.944±9.424 | 2.428±0.071 | 27.783±1.131 |
| 7 | 0.39±0 | 0.75±0.141 | 0.87±0.057 | 0.41±0.041 | 0.593±0.057 | 0.028±0.008 | 23.158±0.679 | 1.755±0.12 | 43.547±4.357 |
| 8 | 0.755±0.346 | 1.065±0.007 | 2.16±0.198 | 0.38±0.003 | 0.508±0.04 | 0.069±0.014 | 37.528±1.579 | 3.28±0.962 | 52.293±8.939 |
| 9 | 0.83±0.014 | 0.75±0.085 | 0.83±0.028 | 0.403±0.05 | 0.528±0.067 | 0.036±0.001 | 22.514±1.455 | 1.989±0.315 | 51.7±0.764 |
| 10 | 0.395±0.049 | 1.16±0.113 | 0.8±0.099 | 0.364±0.006 | 0.523±0.081 | 0.038±0.012 | 25.76±0.976 | 1.252±0.344 | 104.47±1.626 |
|  |  |  |  |  |  |  |  |  |  |
| Resistant clone | 6 | 10 | 8 | 5 | 5 | 8 | 2 | 8 | 5 |
| Sensitive clone | 2 | 5 | 4 | 2 | 1 | 1 | 4 | 5 | 4 |
| Ratio（resistant / sensitive） | 3.3 | 2.1 | 3.6 | 1.3 | 1.9 | 3.5 | 1.8 | 3.2 | 5.3 |
